# Financial Self-Efficacy and Disposition Effect in Investors: The Mediating Role of Versatile Cognitive Style

**DOI:** 10.3389/fpsyg.2018.02705

**Published:** 2019-01-08

**Authors:** Song Tang, Shimin Huang, Jia Zhu, Rui Huang, Zilong Tang, Jianping Hu

**Affiliations:** Laboratory for Behavioral and Regional Finance, Guangdong University of Finance, Guangzhou, China

**Keywords:** financial self-efficacy, disposition effect, versatile cognitive style, decision making, individual differences

## Abstract

The disposition effect refers to the tendency of investors to sell winners too early and hold on to losers too long, which is one of the most documented and robust decision biases. However, few studies have looked beyond demographic and social factors on the disposition effect. The current study investigated the association between financial self-efficacy (FSE) (one’s belief about their personal capability in ultimate financial goals achieving), versatile cognitive style (an individual’s capability in deploying the experiential or rational mode in ways that are contextually appropriate), and the disposition effect. A total of 285 employees from finance-related business completed anonymous questionnaires regarding FSE, rational-experiential inventory, and the disposition effect. Our findings revealed that FSE was significantly and positively associated with versatile cognitive style and the disposition effect. Further, versatile cognitive style partially mediated the relationship between FSE and the disposition effect. Our findings provide valuable guidance for individual investors to make financial decisions based on their characteristics.

## Introduction

The disposition effect refers to investors’ tendency to sell winning stocks too early and keep losing stocks too long (Shefrin and Statman, 1985). It is one of the most widely documented biases in investor behavior. Investors in many countries (i.e., the United States, South Korea, and China) have been shown to exhibit the disposition effect (Odean, 1998; Chen et al., 2007; Choe and Eom, 2009). Considerable body of literature have suggested that the disposition effect has a negative effect on both individuals and markets (Kaustia, 2004; Dhar and Zhu, 2006; Frazzini, 2006; Choe and Eom, 2009; Aspara and Hoffmann, 2015). On the other hand, evolutionary psychology considers decision biases can throw light on the ways humans have evolved to think, helping them achieve satisficing behavior (Sahi, 2017). Thus, more research is needed to understand the psychological mechanisms of the disposition effect.

Previous studies have mainly focused on the influence of demographic characteristics (e.g., age, gender), investors’ preference (e.g., trading frequency) and trading context (e.g., the saliency of information about a stock’s purchase price) on the disposition effect (Taylor and Ogilvie, 1994; Chen et al., 2007; Da Costa et al., 2008). However, to our knowledge, how psychological and cognitive factors contribute to the disposition effect remains largely unexplored. In the current study, we introduce the variable of financial self-efficacy (FSE), which refers to “one’s belief about their capability of organizing and executing courses of action to achieve one’s ultimate financial goals” (Forbes and Kara, 2010). Recent research has found that FSE plays an important role in a series of financial decisions, such as financial inclusion (Mindra and Moya, 2017), financial planning for retirement (Topa et al., 2018), saving behavior (Xiao et al., 2011; Ismail et al., 2017; Magendans et al., 2017), and financial satisfaction (Asebedo and Payne, 2018). However, a few studies have investigated the association between FSE and the disposition effect. For example, Kadous et al. (2014) reported that confidence in trading behavior (a variable near to FSE) was positively associated with the amount of time investors hold losing stock (the lose side of the disposition effect). On the other hand, some empirical studies have found that higher level of FSE is associated with stronger saving intentions and more saving behavior (Xiao et al., 2011; Magendans et al., 2017), suggesting a risk-averse tendency among people with FSE. Taken together, these findings suggest FSE may contribute both to risk-taking in the loss domain and risk-aversion in the gain domain, resulting in greater disposition of selling winning stocks too early and holding losing stocks too long. It also suggests that both sides of the disposition effect may be driven by the same psychological factors (i.e., financial self-efficacy). However, the existing studies examined each side of the disposition effect in isolation (Weber and Welfens, 2008; Kadous et al., 2014), leaving the possible sharing mechanisms underappreciated. This study intends to fill this gap in the literature.

Another gap is that relatively little is known about the mediating mechanisms through which FSE relates to the disposition effect. Based on the social cognitive model, self-efficacy is the foundation of human agency (Bandura, 2001). Among the mechanisms of personal agency, self-efficacy influences a variety of thought patterns including thinking styles (Fuller et al., 2018). Thinking styles refer to individuals’ preferred ways of processing information and dealing with tasks (Zhang and Sternberg, 2005). According to Cognitive-Experiential Self Theory (CEST), there are two types of thinking styles, that is experiential and rational thinking styles (Epstein et al., 1996; Epstein, 1998). The experiential thinking style is fast, automatic, associative, and outcome oriented whereas the rational thinking style is relative slow, effortful, analytical, and process oriented (Phillips et al., 2016). Self-efficacy may cause individuals to integrate experiential and rational thinking styles to fit the varying contingencies (Sagone and De Caroli, 2013), which closely matches the definition of versatile cognitive styles (Sadler-Smith, 2009). In addition, an empirical study conducted by Wolfradt et al. (1999) found individuals with high self-efficacy exhibited higher levels of both rational and experiential processing. Sagone and De Caroli (2013) reported that, individuals with higher scholastic self-efficacy were more likely to adopt various thinking styles. It is suggested that, not only general self-efficacy, but also domain-specific self-efficacy contribute to versatile cognitive styles.

On the other hand, the influence of thinking styles on decision making has received substantial attention (Phillips et al., 2016). However, the findings are complex and sometimes inconsistent. For example, some studies found that individuals only with high experiential thinking exhibited the framing effect (Mahoney et al., 2011; Stark et al., 2017), while another study found that the rational system alone influences the framing effect (Björklund and Bäckström, 2008). In addition, other studies have indicated that the interaction between the rational and experiential preference influences decision making (Shiloh et al., 2002; Ayal et al., 2011). Shiloh et al. (2002) reported that individuals with specific combinations of thinking styles (high rational/high experiential and low rational/low experiential) were the ones most prone to framing effect. It is noticed that the rational and experiential thinking styles were viewed as a dichotomy in these studies. Researchers should discover more rewards in adopting an integrated, organic view of human information processing, that is, versatile cognitive styles (Sadler-Smith, 2009). Thus, based on the integration of the rational and experiential styles, we saw human decision makers as integrated processors, investigating the relationships between versatile cognitive styles and the disposition effect.

Taken together, FSE may be indirectly associated with the disposition effect through versatile cognitive styles. However, no known studies have yet directly examined such mediation model.

The present study extends existing research by empirically examining the association between FSE, versatile cognitive styles, and the disposition effect with two hypotheses. First, we assume that self-efficacy in the financial domain would positively predict the disposition effect (Hypothesis 1); second, we predicted that versatile cognitive styles would mediate the association between FSE and the disposition effect (Hypothesis 2).

## Materials and Methods

### Sample

Participants were employees from finance-related business (i.e., bank, insurance). Employees from securities companies were excluded because they were prohibited to purchase stocks in China. Questionnaires were distributed to employees attending training seminars. They were requested to complete the questionnaires during their coffee breaks and return to the seminar instructors. Sample size was estimated using the G-power 3.1 program. For linear multiple regression, the minimum required number of participants was 263, based on α level of 0.05, power (1-β) of 0.80, effect size (f^2^) of 0.05, and 5 predicting variables (Faul et al., 2009). Considering the potential dropouts and missing data, questionnaires were distributed to 360 participants and 357 were returned. Written informed consent was obtained from all participants. Thirty-nine questionnaires were excluded because of the excessive missing responses (i.e., more than 50%). Participants who were missing data on gender (2.2%), age (2.8%) and education (7.5%) were not included in the analyses. Of the final sample of 285 participants, 163 were males and 122 were females. The mean age of this sample was 31.70 (*SD* = 7.83) with a range of 20–56.

### Measures

#### Financial Self-Efficacy

Participants’ FSE was assessed by FSE scale (Montford and Goldsmith, 2016), which consists of five items. Forward and back-translation procedures were conducted to build the Chinese version of the measurement. Participants were asked to rate each item for their agreement using a seven-point scale ranging from “1 = strongly disagree” to “7 = strongly agree.” The mean was taken, with reverse scoring where necessary. Higher scores represent higher levels of FSE. Cronbach’s α coefficient for the present sample was 0.666.

#### Disposition Effect

Participants’ disposition effect was measured by disposition effect scale, which demonstrated reliability and validity (Zhang, 2012). Participants were asked for their agreement with two statements such as, “Actually, I’m unwilling to sell losing stocks, as compared to winning stocks” on a seven-point scale ranging from “1 = strongly disagree” to “7 = strongly agree.” The mean was taken, with higher scores representing higher levels of disposition effect. Cronbach’s α coefficient for the present sample was 0.628.

#### Versatile Cognitive Style

Versatile cognitive style is the product of the interplay between a rational and an experiential processing (Sadler-Smith, 2009), which were assessed by the short version of Rational Experiential Inventory (REI; Epstein et al., 1996). It consists of 10 items, with 5 items for rational processing and experiential processing, respectively. Forward and back-translation procedures were conducted to build the Chinese version of the measurement. Each item was scored on a five-point scale ranging from “1 = almost always untrue of you” to “5 = almost always true of you.” The mean of each subscale was taken, with reverse scoring where necessary. The Cronbach’s α coefficients of rational processing and experiential processing in this study were 0.65 and 0.80, respectively. According to Sadler-Smith (2009) and Liu et al. (2016), versatile cognitive style was calculated as “(rational + experiential) – ABS (rational – experiential).” A higher score on versatile cognitive style indicates a higher extent to which an individual is able to deploy the rational or experiential mode in ways that are contextually appropriate.

### Data Analysis

First, missing data patterns were assessed using SPSS 22. Little’s MCAR test revealed that data of FSE, rational processing, experiential processing and the disposition effect were missing completely at random (χ^2^ = 452.44, *p* = 0.118). Therefore, missing values were imputed via expectation maximization. Second, preliminary analyses were conducted. Descriptive statistics and bivariate correlations for all variables were presented. Third, SPSS Process Macro was used in the analysis of indirect effect (Hayes, 2017). All continuous variables were standardized. Bootstrapping method (5,000 bootstrap resamples) was used to test the indirect effect, which is an appropriate test of indirect effect and does not assume the normal distribution of scores for given variables. An indirect path is statistically significant if the associated 95% confidence interval (CI; bias corrected) does not include zero.

## Results

### Preliminary Analyses

Means and standard deviations of all variables along with their correlations are presented in Table 1. FSE and versatile cognitive style showed significant and positive correlations with the disposition effect. FSE was positively associated with versatile cognitive style.

**Table 1:** Descriptive statistics and correlations for all variables.

Variables	*M*	*SD*	1	2	3	4	5	6
(1) Gender	0.428	0.500	——					
(2) Age	31.700	7.833	0.009	——				
(3) Education	3.900	0.822	-0.022	-0.265^∗∗∗^	——			
(4) Financial self-efficacy	4.564	0.886	-0.140^∗^	-0.028	0.217^∗∗∗^	——		
(5) Versatile cognitive style	5.897	1.007	-0.090	-0.046	0.128^∗^	0.328^∗∗∗^	——	
(6) Disposition effect	4.460	1.288	-0.057	-0.095	0.077	0.289^∗∗∗^	0.249^∗∗∗^	——


*N = 285. Gender was dummy coded such that 0 = male and 1 = female. Education was coded as 1 = less than primary school and primary school, 2 = high school, 3 = college graduates, 4 = undergraduates, and 5 = master and above. ^∗^p < 0.05 and ^∗∗∗^p < 0.001.*

### Mediation Analyses

**FIGURE 1:**
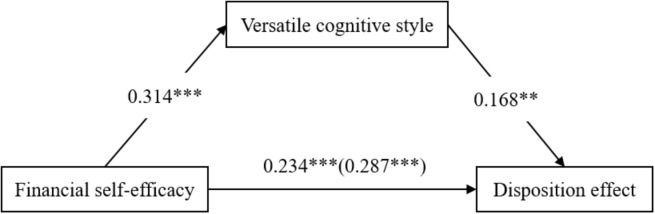
Effect of financial self-efficacy (FSE) and the disposition effect via versatile cognitive style. ^∗∗^*p* < 0.01 and ^∗∗∗^*p* < 0.001.

The Hayes SPSS Process Macro was conducted to examine the mediating effect of versatile cognitive styles on the relationship between FSE and the disposition effect. Results of these analyses are presented in Table 2 and summarized in Figure 1.

**Table 2:** Testing the mediation effect of financial self-efficacy on the disposition effect.

	Criterion: disposition effect			Criterion: versatile cognitive styles			Criterion: disposition effect		
	
Predictors	*b*	*SE*	*t*	*b*	*SE*	*t*	*b*	*SE*	*t*
Gender	-0.033	0.117	-0.284	-0.094	0.117	-0.804	-0.017	0.116	-0.151
Age	-0.090	0.060	-1.506	-0.024	0.060	-0.396	-0.086	0.059	-1.457
Education	-0.009	0.061	-0.147	0.055	0.061	0.893	-0.018	0.060	-0.300
Financial self-efficacy	0.287	0.059	4.858^∗∗∗^	0.314	0.059	5.322^∗∗∗^	0.234	0.061	3.826^∗∗∗^
Versatile cognitive styles							0.168	0.059	2.844^∗∗^
*R*^2^	0.092			0.114			0.117		


*N = 285; ^∗∗^p < 0.01 and ^∗∗∗^p < 0.001.*

First, as seen in Table 2, after controlling for the influence of gender, age and education, FSE had a significant positive effect on the disposition effect (*b* = 0.287, *p* < 0.001). Hypothesis 1 was supported. Second, after controlling for the influence of gender, age, and education, FSE had a significant positive effect on the mediator versatile cognitive styles (*b* = 0.314, *p* < 0.001). Third, when adding versatile cognitive styles into the regression, FSE (*b* = 0.234, *p* < 0.001) and versatile cognitive styles (*b* = 0.168, *p* < 0.01) had significant positive effects on the disposition effect. Further, the Sobel test was statistically significant (*Z* = 2.475, *p* < 0.05). Using 5,000 bootstrap resampling, the lower and upper values of the 95% confidence interval (CI) for the mediating effect of FSE and the disposition effect through versatile cognitive styles (CI = [0.014, 0.113]) did not include zero, demonstrating that the mediating effect were significant. Hypothesis 2 was supported.

## Discussion

The current study extends the literature by looking beyond demographic and social factors, and investigates the association between FSE, versatile cognitive style and the disposition effect. When comparing our results with the literature, we discover some consistent, inconsistent, even surprising findings. The current study contributes to a growing body of literature in the following ways.

First, we found that FSE was positively associated with disposition effect. This finding is consistent with the previous work of Kadous et al. (2014) who found that investors with higher level of confidence in trading behavior hold losing investment longer than those who with lower trading confidence. However, it is noticed that, the comparison between the low and high confidence participants in the study of Kadous et al. (2014) might miss the variance caused by individuals with self-efficacy values that fall in the middle percentile range. Kadous et al. (2014) took the length of time participants held the losing stocks, rather than the differences in the length of time participants held the losing stocks compared to the winning stocks as the index of the disposition effect, leaving the relationship between investment confidence and the disposition effect open to doubt. The current study replicates and extends the findings by maintaining the continuous nature of FSE and operationalizing the disposition effect as the differences in time and willingness to hold the losing stock compared to the winning stocks. In addition, FSE is intrinsically linked to positive outcome expectations (Farrell et al., 2016). It would appear those who high in FSE may have persistent high expectations for their financial outcomes. Selling the winning investments rather than the losing ones are more likely to meet their high financial outcome expectations, resulting in higher levels of disposition effect.

Second, versatile cognitive style was found to partially mediate the relationship between FSE and the disposition effect. At the first stage of the mediation analysis, FSE was associated with higher level of versatile cognitive styles. Self-efficacy is a personality construct derived from social cognitive theory, referring to an estimation of one’s ability to mobile motivation, cognitive resources, and courses of action needed to successfully perform a behavior (Bandura, 1977). It affects how individuals weigh and process information. Self-efficacy has been positively linked to openness to experience (Stajkovic et al., 2018), which has been in turn related to cognitive flexibility (DeYoung et al., 2005). Taken together, we speculate that individuals with higher FSE may more flexibly switch between experiential and rational processing according to the varying contingencies confronting them, that is, in cognitively versatile way that integrate experiential and rational modes of thinking (Hodgkinson and Clarke, 2007). At the second stage of mediation analysis, versatile cognitive styles were positively associated with the disposition effect. Such positive relationship is somewhat surprising. However, it is consistent with the results of Shiloh et al. (2002), who observed that individuals with high rational and high intuitive style combination showed a framing effects. According to CEST, rational and experiential systems operate using different rules (either logical or experiential) to process information, often affecting each other (Toet et al., 2016). A simultaneous approach to both cognitive styles may lead to disinhibit the relationship of each other (Wolfradt et al., 1999; Shiloh et al., 2002; Lechner and Paul, 2017). We speculate that the bimodal preferences may compromise the ability of each other in utilizing rules for information processing, resulting in suboptimal decisions. Adopting an integrated view of cognitive styles, rather than the rational-experiential dichotomy, may provide a new perspective on the mechanism of the disposition effect. Also, the current findings are critical as they challenge the positive impact of FSE and versatile cognitive style. However, as to date there is too little information to explain such pattern of results, these findings should not be overstated and more research is needed to replicate and better understand these results.

The current study had important practical implications. There are noted gender differences in financial behavior, such as financial risk-taking, retirement saving intention and behavior (Xiao et al., 2011; Ismail et al., 2017; Magendans et al., 2017; Panno et al., 2017; Topa et al., 2018). However, there was no gender differences in the disposition effect in the current study. This negative result should be considered preliminary and require confirmation in the future. On the other hand, understanding the causes of the negative result has important practical implications. Lack of gender differences in the disposition effect in the current study may be due to demographics and social characteristics in tested participants. Both male and female participants in the current study were recruited from the same financial seminar with no gender difference in their educational background. These evidences may reflect the similar level of financial knowledge among them, which may lead to decreased gender differences in FSE. Thus, future research aiming to reduce gender differences in financial behaviors may design educational programs to promote gender equality in possession and acquisition of financial literacy, further to boost FSE. In addition, negative effects may show up when investors’ perceived self-efficacy to deal with a financial situation exceeds their actual level of financial knowledge (Moores and Chang, 2009). These educational programs should also allow for a match of FSE toward more accurate self-assessments of financial ability.

Several limitations are acknowledged in this study. First, the cross-sectional design employed in the current study precludes the firm conclusion about the causality or directionality. Testing our mediation model using longitudinal or experimental design may better clarify the directionality between FSE, cognitive style, and the disposition effect. Second, the research is somewhat limited by the exclusive use of self-report measures and may be susceptible to social desirability, selective memory bias, and common method variance problem. It could be substantially improved with multi-method and multi-informant approaches. Third, we recognize that these findings are placed in the context of a single sample of employees from finance-related business. This sample was a convenience sample of participants who were recruited from training seminars. The characteristics of the current sample may differ from other potential samples. It also limited the current findings to be generalized to a larger population. A bigger and wider sample is encouraged to be used in future research. Fourth, this study was tested on a group of Chinese participants. Thus, we should not generalize the current findings to other cultures or geographic locations. It would be more valuable if this model could be conducted in other groups in the future study.

Despite the above limitation, the current study provides empirical evidence for the mediating model in which versatile cognitive style mediates the relationship between FSE and the disposition effect. Our findings also provide valuable guidance for individual investors to make financial decisions based on their characteristics.

## Ethics Statement

This study was carried out in accordance with the recommendations of Ethics Committee of Guangdong University of Finance. The protocol was approved by Guangdong University of Finance Human Investigation Committee.

## Author Contributions

SH, ST, and JH conceived and designed the research. ST, JZ, RH, and ZT performed the research. SH, RH, and ZT analyzed the data. ST, SH, JZ, and JH contributed to the writing of the manuscript.

## Conflict of Interest Statement

The authors declare that the research was conducted in the absence of any commercial or financial relationships that could be construed as a potential conflict of interest.
